# Hexaaqua­cobalt(II) bis­(2,2′-sulfanediyldiacetato-κ^3^ *O*,*S*,*O*′)cobaltate(II) tetra­hydrate

**DOI:** 10.1107/S1600536811040979

**Published:** 2011-10-12

**Authors:** Huang Wang, Shan Gao, Seik Weng Ng

**Affiliations:** a Key Laboratory of Functional Inorganic Material Chemistry, Ministry of Education, Heilongjiang University, Harbin 150080, People’s Republic of China; b Department of Chemistry, University of Malaya, 50603 Kuala Lumpur, Malaysia; c Chemistry Department, Faculty of Science, King Abdulaziz University, PO Box 80203 Jeddah, Saudi Arabia

## Abstract

The two Co^II^ atoms in the title salt, [Co(H_2_O)_6_][Co(C_4_H_4_O_4_S)_2_]·4H_2_O, exist in an octa­hedral coordination environment. In the cation, the Co atom is surrounded by six water mol­ecules, and in the anion, it is *bis*-*O*,*S*,*O*′-chelated by the thio­acetate ligands. The cations, anions and uncoordinated water mol­ecules are linked by O—H⋯O hydrogen bonds into a three-dimensional network.

## Related literature

For the isotypic nickel(II) analog, see: Pan *et al.* (2005 ▶).
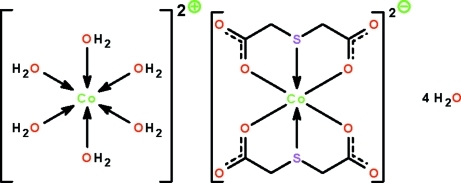


## Experimental

### 

#### Crystal data

[Co(H_2_O)_6_][Co(C_4_H_4_O_4_S)_2_]·4H_2_O
*M* *_r_* = 594.28
Monoclinic, 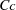

*a* = 18.8627 (9) Å
*b* = 13.5779 (7) Å
*c* = 8.9535 (4) Å
β = 101.403 (1)°
*V* = 2247.87 (19) Å^3^
*Z* = 4
Mo *K*α radiation
μ = 1.74 mm^−1^
*T* = 293 K
0.18 × 0.14 × 0.14 mm

#### Data collection

Rigaku R-AXIS RAPID IP diffractometer
Absorption correction: multi-scan (*ABSCOR*; Higashi, 1995 ▶) *T*_min_ = 0.745, *T*_max_ = 0.793
10837 measured reflections
4978 independent reflections
4802 reflections with *I* > 2σ(*I*)
*R*_int_ = 0.025

#### Refinement

*R*[*F* ^2^ > 2σ(*F* ^2^)] = 0.026
*wR*(*F* ^2^) = 0.067
*S* = 1.01
4978 reflections
331 parameters
56 restraints
H atoms treated by a mixture of independent and constrained refinement
Δρ_max_ = 0.74 e Å^−3^
Δρ_min_ = −0.58 e Å^−3^
Absolute structure: Flack (1983 ▶), 2402 Friedel pairs
Flack parameter: 0.02 (1)

### 

Data collection: *RAPID-AUTO* (Rigaku, 1998 ▶); cell refinement: *RAPID-AUTO*; data reduction: *CrystalClear* (Rigaku/MSC, 2002 ▶); program(s) used to solve structure: *SHELXS97* (Sheldrick, 2008 ▶); program(s) used to refine structure: *SHELXL97* (Sheldrick, 2008 ▶); molecular graphics: *X-SEED* (Barbour, 2001 ▶); software used to prepare material for publication: *publCIF* (Westrip, 2010 ▶).

## Supplementary Material

Crystal structure: contains datablock(s) global, I. DOI: 10.1107/S1600536811040979/bt5666sup1.cif

Structure factors: contains datablock(s) I. DOI: 10.1107/S1600536811040979/bt5666Isup2.hkl

Additional supplementary materials:  crystallographic information; 3D view; checkCIF report

## Figures and Tables

**Table 1:** Hydrogen-bond geometry (Å, °)

*D*—H⋯*A*	*D*—H	H⋯*A*	*D*⋯*A*	*D*—H⋯*A*
O1w—H11⋯O2	0.84 (1)	1.89 (2)	2.707 (4)	162 (5)
O1w—H12⋯O6^i^	0.84 (1)	1.95 (1)	2.791 (4)	173 (5)
O2w—H21⋯O8w^ii^	0.84 (1)	2.08 (2)	2.824 (3)	147 (3)
O2w—H22⋯O4^iii^	0.85 (1)	1.98 (1)	2.813 (3)	170 (4)
O3w—H31⋯O4^iv^	0.83 (1)	1.87 (2)	2.671 (3)	163 (5)
O3w—H32⋯O8^v^	0.83 (1)	1.85 (2)	2.666 (3)	168 (5)
O4w—H41⋯O7w^vi^	0.85 (1)	2.06 (2)	2.880 (4)	162 (4)
O4w—H42⋯O8^vii^	0.85 (1)	1.96 (1)	2.805 (3)	173 (4)
O5w—H51⋯O9w^iii^	0.83 (1)	1.84 (2)	2.657 (4)	166 (3)
O5w—H52⋯O5^i^	0.84 (1)	1.89 (1)	2.721 (3)	179 (5)
O6w—H61⋯O1	0.83 (1)	1.91 (2)	2.726 (3)	166 (4)
O6w—H62⋯O10w	0.84 (1)	1.91 (1)	2.746 (3)	177 (4)
O7w—H71⋯O2	0.83 (1)	2.18 (4)	2.828 (4)	135 (5)
O7w—H72⋯O8w	0.84 (1)	1.96 (2)	2.777 (4)	165 (5)
O8w—H81⋯O6^i^	0.85 (1)	1.91 (1)	2.751 (3)	172 (5)
O8w—H82⋯O3^viii^	0.84 (1)	2.13 (1)	2.965 (3)	168 (4)
O9w—H91⋯O3	0.84 (1)	2.08 (5)	2.797 (4)	142 (8)
O9w—H92⋯O10w	0.85 (1)	2.12 (8)	2.759 (5)	132 (9)
O10w—H101⋯O7w^vi^	0.84 (1)	2.02 (2)	2.831 (4)	162 (4)
O10w—H102⋯O7^vii^	0.84 (1)	1.93 (2)	2.701 (3)	152 (4)

Symmetry codes: (i) 
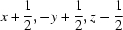
; (ii) 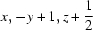; (iii) 
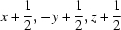
; (iv) 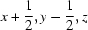; (v) 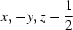; (vi) 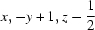; (vii) 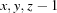; (viii) 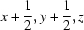.

**Fig. 1.**
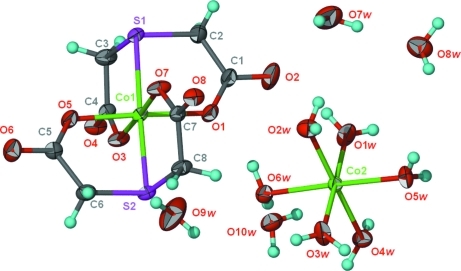
Anisotropic displacement ellipsoid plot (Barbour, 2001) of Co(H2O)62+.Co(C4H4O4S)2–.4H2O at the 50% probability level; hydrogen atoms are drawn as spheres of arbitrary radius.

## supplementary crystallographic information

### Comment

First-row transition metal dications form a plethora of metal dicarboxylates; in some cases, a a direct metal–carboxylate bond is formed and in other cases, the product consists of hexaaquametal cations and carboxylate ions, the anion interacting indirectly in an outer-sphere type of coordination. Thioacetic acid yields several metal carboxylates; the reaction of the deprotonated acid with cobalt(II) ions gives the hexaaquacobalt(II) di(carboxylato)cobaltate(II) (Scheme I, Fig. 1). The two Co^II^ atoms in the salt exist in octahedral coordination environments. That in the cation is surrounded by water water molecules; that in the anion is O,*S*,*O*'-chelated by the thioacetate ligands. The cations, anions and lattice water molecules are linked by O···H···O into a three-dimensional network (Table 1). The salt is isostructural with the nickel(II) analog (Pan *et al.*, 2005).

### Experimental

Cobalt diacetate (1 mmol) was added to an aqueous solution of thiodiacetic acid acid (1 mmol) that was earlier been treated with 1*M* sodium hydroxide to a pH of 6. The filtered solution was set aside for several days, after which pink prismatic crystals separated from solution.

### Refinement

Carbon-bound H-atoms were placed in calculated positions (C–H 0.93 Å) and were included in the refinement in the riding model approximation, with *U*(H) set to 1.2*U*(C). The water H-atoms were located in a difference Fourier map, and were refined with distance restraints of O–H 0.84±0.01 Å and H···H 1.37±0.01 Å; their U values were set to 1.5U_eq_(O).

The anisotropic displacement ellipsoids of the lattice water O atoms were restrained to be nearly isotropic.

The (5 9 9), (-5 9 - 9) (9 9 8) and (9 3 - 11) reflections were omitted owing to bad agreement.

### Crystal data

**Table:**

[Co(H_2_O)_6_][Co(C_4_H_4_O_4_S)_2_]·4H_2_O	*F*(000) = 1224
*M**_r_* = 594.28	*D*_x_ = 1.756 Mg m^−^^3^
Monoclinic, *C**c*	Mo *K*α radiation, λ = 0.71073 Å
Hall symbol: C -2yc	Cell parameters from 10500 reflections
*a* = 18.8627 (9) Å	θ = 3.0–27.5°
*b* = 13.5779 (7) Å	µ = 1.74 mm^−^^1^
*c* = 8.9535 (4) Å	*T* = 293 K
β = 101.403 (1)°	Prism, pink
*V* = 2247.87 (19) Å^3^	0.18 × 0.14 × 0.14 mm
*Z* = 4	

### Data collection

**Table:**

Rigaku R-AXIS RAPID IP diffractometer	4978 independent reflections
Radiation source: fine-focus sealed tube	4802 reflections with *I* > 2σ(*I*)
graphite	*R*_int_ = 0.025
ω scans	θ_max_ = 27.5°, θ_min_ = 3.0°
Absorption correction: multi-scan (*ABSCOR*; Higashi, 1995)	*h* = −24→24
*T*_min_ = 0.745, *T*_max_ = 0.793	*k* = −17→17
10837 measured reflections	*l* = −11→11

### Refinement

**Table:**

Refinement on *F*^2^	Secondary atom site location: difference Fourier map
Least-squares matrix: full	Hydrogen site location: inferred from neighbouring sites
*R*[*F*^2^ > 2σ(*F*^2^)] = 0.026	H atoms treated by a mixture of independent and constrained refinement
*wR*(*F*^2^) = 0.067	*w* = 1/[σ^2^(*F*_o_^2^) + (0.0456*P*)^2^] where *P* = (*F*_o_^2^ + 2*F*_c_^2^)/3
*S* = 1.01	(Δ/σ)_max_ = 0.001
4978 reflections	Δρ_max_ = 0.74 e Å^−^^3^
331 parameters	Δρ_min_ = −0.58 e Å^−^^3^
56 restraints	Absolute structure: Flack (1983), 2402 Friedel pairs
Primary atom site location: structure-invariant direct methods	Flack parameter: 0.02 (1)

### Fractional atomic coordinates and isotropic or equivalent isotropic displacement parameters (Å^2^)

**Table:**

	*x*	*y*	*z*	*U*_iso_*/*U*_eq_	
Co1	0.500004 (19)	0.23844 (2)	0.50001 (4)	0.02113 (8)	
Co2	0.756568 (19)	0.19476 (2)	0.24733 (4)	0.02289 (8)	
S1	0.49039 (3)	0.40580 (5)	0.60363 (7)	0.02473 (13)	
S2	0.51526 (3)	0.06991 (5)	0.40430 (7)	0.02585 (14)	
O1	0.58222 (12)	0.30010 (14)	0.4126 (3)	0.0343 (5)	
O2	0.65276 (15)	0.4269 (2)	0.3849 (4)	0.0643 (9)	
O3	0.42627 (11)	0.29204 (14)	0.3183 (2)	0.0302 (4)	
O4	0.35388 (11)	0.40998 (15)	0.2070 (3)	0.0346 (5)	
O5	0.41625 (11)	0.17334 (15)	0.5790 (3)	0.0328 (4)	
O6	0.35800 (12)	0.04251 (16)	0.6378 (3)	0.0415 (5)	
O7	0.57090 (12)	0.18662 (14)	0.6866 (2)	0.0335 (5)	
O8	0.65760 (11)	0.08440 (14)	0.7929 (2)	0.0317 (4)	
O1W	0.75186 (17)	0.34735 (15)	0.2392 (4)	0.0485 (5)	
H11	0.728 (2)	0.380 (3)	0.293 (5)	0.073*	
H12	0.782 (2)	0.385 (3)	0.211 (5)	0.073*	
O2W	0.75883 (13)	0.19813 (16)	0.4850 (3)	0.0376 (5)	
H21	0.771 (2)	0.2564 (10)	0.510 (4)	0.056*	
H22	0.7864 (19)	0.1600 (19)	0.545 (4)	0.056*	
O3W	0.75775 (13)	0.04584 (14)	0.2516 (4)	0.0444 (5)	
H31	0.7904 (18)	0.013 (3)	0.226 (6)	0.067*	
H32	0.7261 (18)	0.010 (3)	0.276 (6)	0.067*	
O4W	0.75304 (12)	0.19686 (15)	0.0065 (3)	0.0348 (5)	
H41	0.751 (2)	0.2540 (13)	−0.033 (4)	0.052*	
H42	0.7221 (18)	0.162 (2)	−0.053 (4)	0.052*	
O5W	0.86746 (11)	0.19782 (15)	0.2686 (3)	0.0348 (5)	
H51	0.8928 (18)	0.201 (3)	0.3561 (19)	0.052*	
H52	0.883 (2)	0.237 (3)	0.210 (3)	0.052*	
O6W	0.64320 (11)	0.19108 (14)	0.2156 (3)	0.0309 (4)	
H61	0.631 (2)	0.223 (3)	0.287 (3)	0.046*	
H62	0.629 (2)	0.221 (2)	0.133 (2)	0.046*	
O7W	0.71149 (19)	0.6167 (2)	0.3620 (4)	0.0693 (8)	
H71	0.712 (3)	0.566 (3)	0.414 (6)	0.104*	
H72	0.746 (2)	0.616 (4)	0.314 (5)	0.104*	
O8W	0.81452 (15)	0.64657 (18)	0.1837 (3)	0.0516 (6)	
H81	0.832 (2)	0.5909 (17)	0.168 (6)	0.077*	
H82	0.8486 (16)	0.682 (3)	0.231 (5)	0.077*	
O9W	0.45875 (19)	0.2664 (5)	0.0292 (4)	0.1126 (15)	
H91	0.456 (5)	0.247 (6)	0.117 (4)	0.169*	
H92	0.494 (4)	0.237 (7)	0.002 (9)	0.169*	
O10W	0.59157 (12)	0.2904 (2)	−0.0509 (3)	0.0421 (5)	
H101	0.622 (2)	0.329 (2)	−0.077 (4)	0.063*	
H102	0.585 (2)	0.242 (2)	−0.111 (4)	0.063*	
C1	0.60292 (15)	0.3899 (2)	0.4351 (4)	0.0316 (6)	
C2	0.56550 (18)	0.4557 (2)	0.5315 (4)	0.0398 (7)	
H2A	0.5485	0.5136	0.4716	0.048*	
H2B	0.6015	0.4777	0.6179	0.048*	
C3	0.41322 (17)	0.4397 (2)	0.4608 (4)	0.0353 (7)	
H3A	0.3710	0.4386	0.5074	0.042*	
H3B	0.4198	0.5071	0.4307	0.042*	
C4	0.39701 (13)	0.37693 (18)	0.3173 (3)	0.0247 (5)	
C5	0.40418 (15)	0.0819 (2)	0.5766 (3)	0.0276 (5)	
C6	0.44673 (16)	0.0137 (2)	0.4915 (3)	0.0318 (6)	
H6A	0.4126	−0.0197	0.4124	0.038*	
H6B	0.4698	−0.0362	0.5622	0.038*	
C7	0.60916 (13)	0.11060 (19)	0.6845 (3)	0.0248 (5)	
C8	0.59746 (16)	0.0475 (2)	0.5424 (3)	0.0321 (6)	
H8A	0.5978	−0.0210	0.5731	0.039*	
H8B	0.6382	0.0572	0.4925	0.039*	

### Atomic displacement parameters (Å^2^)

**Table:**

	*U*^11^	*U*^22^	*U*^33^	*U*^12^	*U*^13^	*U*^23^
Co1	0.02393 (15)	0.01925 (16)	0.02064 (14)	0.00472 (13)	0.00543 (11)	0.00147 (13)
Co2	0.02136 (14)	0.02034 (16)	0.02750 (15)	0.00004 (12)	0.00615 (12)	0.00000 (13)
S1	0.0264 (3)	0.0259 (3)	0.0225 (3)	0.0038 (2)	0.0062 (2)	−0.0031 (2)
S2	0.0306 (3)	0.0268 (3)	0.0203 (3)	0.0052 (2)	0.0051 (2)	−0.0030 (2)
O1	0.0363 (10)	0.0275 (10)	0.0447 (11)	0.0001 (8)	0.0219 (9)	−0.0066 (8)
O2	0.0666 (17)	0.0410 (14)	0.104 (2)	−0.0081 (12)	0.0631 (18)	−0.0073 (14)
O3	0.0382 (10)	0.0246 (9)	0.0259 (9)	0.0114 (8)	0.0019 (8)	−0.0031 (7)
O4	0.0375 (11)	0.0308 (10)	0.0320 (11)	0.0121 (8)	−0.0018 (9)	−0.0002 (8)
O5	0.0366 (10)	0.0242 (9)	0.0430 (11)	0.0008 (8)	0.0213 (9)	−0.0030 (9)
O6	0.0427 (11)	0.0304 (11)	0.0578 (14)	−0.0002 (9)	0.0252 (10)	0.0057 (10)
O7	0.0432 (11)	0.0285 (10)	0.0254 (9)	0.0162 (8)	−0.0017 (8)	−0.0049 (8)
O8	0.0317 (10)	0.0308 (10)	0.0290 (11)	0.0083 (8)	−0.0026 (8)	−0.0004 (8)
O1W	0.0592 (13)	0.0229 (10)	0.0758 (16)	−0.0026 (11)	0.0435 (12)	−0.0015 (12)
O2W	0.0467 (12)	0.0348 (11)	0.0289 (10)	0.0014 (9)	0.0018 (10)	0.0009 (8)
O3W	0.0284 (9)	0.0185 (9)	0.0899 (16)	0.0008 (8)	0.0198 (10)	−0.0002 (12)
O4W	0.0392 (11)	0.0359 (12)	0.0292 (10)	−0.0013 (8)	0.0064 (9)	−0.0026 (8)
O5W	0.0235 (9)	0.0429 (12)	0.0384 (12)	−0.0063 (8)	0.0072 (9)	0.0058 (9)
O6W	0.0275 (10)	0.0327 (10)	0.0327 (10)	0.0042 (8)	0.0065 (9)	−0.0027 (8)
O7W	0.087 (2)	0.0499 (15)	0.078 (2)	0.0073 (15)	0.0313 (17)	−0.0139 (14)
O8W	0.0566 (14)	0.0336 (12)	0.0609 (16)	0.0021 (10)	0.0030 (12)	−0.0041 (11)
O9W	0.0498 (17)	0.246 (4)	0.0406 (16)	0.022 (2)	0.0068 (15)	−0.022 (2)
O10W	0.0409 (11)	0.0512 (13)	0.0338 (11)	0.0057 (10)	0.0065 (10)	−0.0110 (10)
C1	0.0323 (14)	0.0249 (13)	0.0419 (16)	0.0005 (11)	0.0180 (13)	−0.0007 (11)
C2	0.0387 (15)	0.0305 (15)	0.055 (2)	−0.0056 (12)	0.0212 (14)	−0.0114 (14)
C3	0.0349 (14)	0.0317 (14)	0.0363 (16)	0.0129 (11)	0.0000 (13)	−0.0073 (12)
C4	0.0259 (12)	0.0232 (11)	0.0253 (12)	0.0035 (9)	0.0061 (10)	0.0003 (10)
C5	0.0295 (12)	0.0253 (12)	0.0275 (13)	0.0035 (10)	0.0041 (11)	0.0021 (10)
C6	0.0416 (15)	0.0213 (12)	0.0339 (14)	0.0011 (10)	0.0112 (12)	−0.0018 (10)
C7	0.0238 (11)	0.0230 (11)	0.0276 (13)	0.0032 (9)	0.0053 (11)	0.0000 (10)
C8	0.0319 (13)	0.0335 (14)	0.0282 (14)	0.0124 (11)	−0.0007 (11)	−0.0077 (11)

### Geometric parameters (Å, °)

**Table:**

Co1—O7	2.0472 (19)	O3W—H31	0.831 (11)
Co1—O1	2.049 (2)	O3W—H32	0.830 (11)
Co1—O3	2.0532 (19)	O4W—H41	0.851 (11)
Co1—O5	2.054 (2)	O4W—H42	0.850 (11)
Co1—S1	2.4746 (7)	O5W—H51	0.834 (11)
Co1—S2	2.4801 (7)	O5W—H52	0.835 (11)
Co2—O3W	2.0224 (19)	O6W—H61	0.834 (11)
Co2—O5W	2.063 (2)	O6W—H62	0.840 (11)
Co2—O1W	2.075 (2)	O7W—H71	0.834 (11)
Co2—O6W	2.101 (2)	O7W—H72	0.841 (11)
Co2—O2W	2.120 (2)	O8W—H81	0.847 (11)
Co2—O4W	2.145 (2)	O8W—H82	0.844 (11)
S1—C3	1.797 (3)	O9W—H91	0.843 (11)
S1—C2	1.802 (3)	O9W—H92	0.845 (11)
S2—C8	1.807 (3)	O10W—H101	0.843 (11)
S2—C6	1.806 (3)	O10W—H102	0.839 (11)
O1—C1	1.283 (3)	C1—C2	1.511 (4)
O2—C1	1.227 (4)	C2—H2A	0.9700
O3—C4	1.277 (3)	C2—H2B	0.9700
O4—C4	1.233 (3)	C3—C4	1.522 (4)
O5—C5	1.261 (4)	C3—H3A	0.9700
O6—C5	1.238 (4)	C3—H3B	0.9700
O7—C7	1.262 (3)	C5—C6	1.525 (4)
O8—C7	1.246 (3)	C6—H6A	0.9700
O1W—H11	0.843 (11)	C6—H6B	0.9700
O1W—H12	0.843 (11)	C7—C8	1.514 (4)
O2W—H21	0.843 (11)	C8—H8A	0.9700
O2W—H22	0.845 (11)	C8—H8B	0.9700
			
O7—Co1—O1	91.74 (10)	Co2—O4W—H41	115 (3)
O7—Co1—O3	177.79 (10)	Co2—O4W—H42	120 (3)
O1—Co1—O3	89.88 (9)	H41—O4W—H42	106 (2)
O7—Co1—O5	89.64 (10)	Co2—O5W—H51	118 (3)
O1—Co1—O5	177.55 (10)	Co2—O5W—H52	115 (3)
O3—Co1—O5	88.79 (9)	H51—O5W—H52	111 (2)
O7—Co1—S1	95.40 (6)	Co2—O6W—H61	108 (3)
O1—Co1—S1	83.40 (6)	Co2—O6W—H62	104 (3)
O3—Co1—S1	83.29 (5)	H61—O6W—H62	110 (2)
O5—Co1—S1	98.49 (6)	H71—O7W—H72	111 (5)
O7—Co1—S2	82.13 (5)	H81—O8W—H82	108 (2)
O1—Co1—S2	95.56 (6)	H91—O9W—H92	109 (9)
O3—Co1—S2	99.21 (6)	H101—O10W—H102	109 (2)
O5—Co1—S2	82.63 (6)	O2—C1—O1	124.3 (3)
S1—Co1—S2	177.30 (3)	O2—C1—C2	116.4 (3)
O3W—Co2—O5W	90.64 (9)	O1—C1—C2	119.3 (3)
O3W—Co2—O1W	178.12 (12)	C1—C2—S1	118.1 (2)
O5W—Co2—O1W	91.07 (10)	C1—C2—H2A	107.8
O3W—Co2—O6W	89.20 (8)	S1—C2—H2A	107.8
O5W—Co2—O6W	177.57 (10)	C1—C2—H2B	107.8
O1W—Co2—O6W	89.06 (10)	S1—C2—H2B	107.8
O3W—Co2—O2W	90.26 (12)	H2A—C2—H2B	107.1
O5W—Co2—O2W	95.05 (9)	C4—C3—S1	117.2 (2)
O1W—Co2—O2W	90.35 (11)	C4—C3—H3A	108.0
O6W—Co2—O2W	87.38 (9)	S1—C3—H3A	108.0
O3W—Co2—O4W	91.75 (12)	C4—C3—H3B	108.0
O5W—Co2—O4W	85.52 (9)	S1—C3—H3B	108.0
O1W—Co2—O4W	87.63 (11)	H3A—C3—H3B	107.2
O6W—Co2—O4W	92.06 (9)	O4—C4—O3	123.4 (2)
O2W—Co2—O4W	177.91 (8)	O4—C4—C3	117.6 (2)
C3—S1—C2	103.43 (17)	O3—C4—C3	119.0 (2)
C3—S1—Co1	94.45 (10)	O6—C5—O5	123.9 (3)
C2—S1—Co1	95.16 (10)	O6—C5—C6	116.4 (2)
C8—S2—C6	102.94 (15)	O5—C5—C6	119.7 (3)
C8—S2—Co1	93.51 (9)	C5—C6—S2	116.86 (19)
C6—S2—Co1	95.67 (9)	C5—C6—H6A	108.1
C1—O1—Co1	123.92 (19)	S2—C6—H6A	108.1
C4—O3—Co1	123.34 (17)	C5—C6—H6B	108.1
C5—O5—Co1	124.48 (19)	S2—C6—H6B	108.1
C7—O7—Co1	123.53 (18)	H6A—C6—H6B	107.3
Co2—O1W—H11	122 (4)	O8—C7—O7	123.7 (3)
Co2—O1W—H12	126 (4)	O8—C7—C8	117.1 (2)
H11—O1W—H12	108 (2)	O7—C7—C8	119.1 (2)
Co2—O2W—H21	104 (3)	C7—C8—S2	116.28 (18)
Co2—O2W—H22	121 (3)	C7—C8—H8A	108.2
H21—O2W—H22	108 (2)	S2—C8—H8A	108.2
Co2—O3W—H31	123 (3)	C7—C8—H8B	108.2
Co2—O3W—H32	126 (3)	S2—C8—H8B	108.2
H31—O3W—H32	111 (2)	H8A—C8—H8B	107.4
			
O7—Co1—S1—C3	−167.18 (14)	O1—Co1—O7—C7	−78.5 (2)
O1—Co1—S1—C3	101.68 (14)	O5—Co1—O7—C7	99.5 (2)
O3—Co1—S1—C3	11.02 (13)	S1—Co1—O7—C7	−162.0 (2)
O5—Co1—S1—C3	−76.74 (14)	S2—Co1—O7—C7	16.9 (2)
O7—Co1—S1—C2	88.87 (14)	Co1—O1—C1—O2	178.9 (3)
O1—Co1—S1—C2	−2.26 (14)	Co1—O1—C1—C2	0.1 (4)
O3—Co1—S1—C2	−92.93 (14)	O2—C1—C2—S1	178.4 (3)
O5—Co1—S1—C2	179.32 (14)	O1—C1—C2—S1	−2.7 (4)
O7—Co1—S2—C8	−17.84 (13)	C3—S1—C2—C1	−92.7 (3)
O1—Co1—S2—C8	73.15 (13)	Co1—S1—C2—C1	3.2 (3)
O3—Co1—S2—C8	163.94 (13)	C2—S1—C3—C4	79.5 (3)
O5—Co1—S2—C8	−108.50 (13)	Co1—S1—C3—C4	−16.9 (3)
O7—Co1—S2—C6	85.55 (12)	Co1—O3—C4—O4	178.4 (2)
O1—Co1—S2—C6	176.55 (12)	Co1—O3—C4—C3	−4.2 (4)
O3—Co1—S2—C6	−92.67 (12)	S1—C3—C4—O4	−165.9 (2)
O5—Co1—S2—C6	−5.11 (12)	S1—C3—C4—O3	16.6 (4)
O7—Co1—O1—C1	−93.6 (2)	Co1—O5—C5—O6	172.7 (2)
O3—Co1—O1—C1	84.9 (2)	Co1—O5—C5—C6	−8.8 (4)
S1—Co1—O1—C1	1.6 (2)	O6—C5—C6—S2	−179.0 (2)
S2—Co1—O1—C1	−175.9 (2)	O5—C5—C6—S2	2.3 (4)
O1—Co1—O3—C4	−89.2 (2)	C8—S2—C6—C5	98.2 (2)
O5—Co1—O3—C4	92.9 (2)	Co1—S2—C6—C5	3.3 (2)
S1—Co1—O3—C4	−5.8 (2)	Co1—O7—C7—O8	172.3 (2)
S2—Co1—O3—C4	175.2 (2)	Co1—O7—C7—C8	−6.5 (4)
O7—Co1—O5—C5	−73.5 (2)	O8—C7—C8—S2	166.7 (2)
O3—Co1—O5—C5	108.1 (2)	O7—C7—C8—S2	−14.4 (4)
S1—Co1—O5—C5	−168.9 (2)	C6—S2—C8—C7	−75.0 (3)
S2—Co1—O5—C5	8.6 (2)	Co1—S2—C8—C7	21.6 (2)

### Hydrogen-bond geometry (Å, °)

**Table:**

*D*—H···*A*	*D*—H	H···*A*	*D*···*A*	*D*—H···*A*
O1w—H11···O2	0.84 (1)	1.89 (2)	2.707 (4)	162 (5)
O1w—H12···O6^i^	0.84 (1)	1.95 (1)	2.791 (4)	173 (5)
O2w—H21···O8w^ii^	0.84 (1)	2.08 (2)	2.824 (3)	147 (3)
O2w—H22···O4^iii^	0.85 (1)	1.98 (1)	2.813 (3)	170 (4)
O3w—H31···O4^iv^	0.83 (1)	1.87 (2)	2.671 (3)	163 (5)
O3w—H32···O8^v^	0.83 (1)	1.85 (2)	2.666 (3)	168 (5)
O4w—H41···O7w^vi^	0.85 (1)	2.06 (2)	2.880 (4)	162 (4)
O4w—H42···O8^vii^	0.85 (1)	1.96 (1)	2.805 (3)	173 (4)
O5w—H51···O9w^iii^	0.83 (1)	1.84 (2)	2.657 (4)	166 (3)
O5w—H52···O5^i^	0.84 (1)	1.89 (1)	2.721 (3)	179 (5)
O6w—H61···O1	0.83 (1)	1.91 (2)	2.726 (3)	166 (4)
O6w—H62···O10w	0.84 (1)	1.91 (1)	2.746 (3)	177 (4)
O7w—H71···O2	0.83 (1)	2.18 (4)	2.828 (4)	135 (5)
O7w—H72···O8w	0.84 (1)	1.96 (2)	2.777 (4)	165 (5)
O8w—H81···O6^i^	0.85 (1)	1.91 (1)	2.751 (3)	172 (5)
O8w—H82···O3^viii^	0.84 (1)	2.13 (1)	2.965 (3)	168 (4)
O9w—H91···O3	0.84 (1)	2.08 (5)	2.797 (4)	142 (8)
O9w—H92···O10w	0.85 (1)	2.12 (8)	2.759 (5)	132 (9)
O10w—H101···O7w^vi^	0.84 (1)	2.02 (2)	2.831 (4)	162 (4)
O10w—H102···O7^vii^	0.84 (1)	1.93 (2)	2.701 (3)	152 (4)

Symmetry codes: (i) *x*+1/2, −*y*+1/2, *z*−1/2; (ii) *x*, −*y*+1, *z*+1/2; (iii) *x*+1/2, −*y*+1/2, *z*+1/2; (iv) *x*+1/2, *y*−1/2, *z*; (v) *x*, −*y*, *z*−1/2; (vi) *x*, −*y*+1, *z*−1/2; (vii) *x*, *y*, *z*−1; (viii) *x*+1/2, *y*+1/2, *z*.
